# ATM phosphorylation of CD98HC increases antiporter membrane localization and prevents chronic toxic glutamate accumulation in Ataxia telangiectasia

**DOI:** 10.21203/rs.3.rs-4947457/v1

**Published:** 2024-09-05

**Authors:** Alexander Bishop, July Carolina Romero, Sonal Tonapi, Manish Parihar, Eva Loranc, Henry Miller, Liesl Lawrence, Nicklas Bassani, Daniel Robledo, Lin Cao, Jia Nie, Kairi Kanda, Aiola Stoja, Natalia Garcia, Aparna Gorthi, Brian Stoveken, Andrew Lane, Teresa Fan, Teresa Cassel, Shan Zha, Nicolas Musi

**Affiliations:** University of Texas Health at San Antonio; University of Texas Health at San Antonio; University of Texas Health at San Antonio; University of Texas Health at San Antonio; University of Texas Health at San Antonio; University of Texas Health at San Antonio; University of Texas Health at San Antonio; University of Texas Health at San Antonio; University of Texas Health at San Antonio; University of Texas Health at San Antonio; University of Texas Health at San Antonio; University of Texas Health at San Antonio; University of Texas Health at San Antonio; University of Texas Health at San Antonio; University of Texas Health at San Antonio; University of Texas Health at San Antonio; University of Kentucky College of Medicine; Center for Environmental and Systems Biochemistry, University of Kentucky; Markey Cancer Center, University of Kentucky; Columbia University; University of California

## Abstract

Ataxia telangiectasia (A-T) is a rare genetic disorder characterized by neurological defects, immunodeficiency, cancer predisposition, radiosensitivity, decreased blood vessel integrity, and diabetes. ATM, the protein mutated in A-T, responds to DNA damage and oxidative stress, but its functional relationship to the progressive clinical manifestation of A-T is not understood. CD98HC chaperones cystine/glutamate (x_c_^−^) and cationic/neutral amino acid (y^+^L) antiporters to the cell membrane, and CD98HC phosphorylation by ATM accelerates membrane localization to acutely increase amino acid transport. Loss of ATM impacts tissues reliant on SLC family antiporters relevant to A-T phenotypes, such as endothelial cells (telangiectasia) and pancreatic α-cells (fatty liver and diabetes) with toxic glutamate accumulation. Bypassing the antiporters restores intracellular metabolic balance both in ATM-deficient cells and mouse models. These findings provide new insight into the long-known benefits of N-acetyl cysteine to A-T cells beyond oxidative stress through removing excess glutamate by production of glutathione.

## Introduction

Ataxia telangiectasia (A-T) is a rare recessive genetic disorder resulting from the mutation, and usually loss of protein, of ATM^1^. A-T progressively manifests in multiple phenotypes including immunodeficiency, neurological defects, cancer, telangiectasia, and diabetes without any established treatment^2^. ATM, a phosphatidylinositol 3-kinase-like kinase, is largely nuclear in location, though a measurable proportion is found in the cytoplasm^3,4^. Most work to date has outlined a role for ATM in orchestrating cellular response to DNA double-strand break and oxidative stress^5^. In addition to autophosphorylation, ATM phosphorylates and activates key proteins including p53^6^ and checkpoint kinase CHK2^7^. As such, in response to damage, ATM regulates cell cycle progression, apoptosis, and DNA repair processes. Beyond these functions, ATM regulates peroxisome and mitochondrial function^8,9^ and impacts glucose import (GLUT1 pS490) and glycolysis/pentose phosphate pathway (PPP) switch (HSP27:G6PD), particularly in response to exogenous damage^10,11^.

Tissues of *Atm*-null mice display increased levels of oxidative stress and damage^12^. These observations fit prior work in *Atm*^*−/−*^ mice demonstrating that treatment with the anti-oxidant N-acetyl cysteine (NAC) increased survival, delayed lymphoma onset, and reduced genomic instability^13,14^. NAC increases intracellular cysteine and augments the production of glutathione (GSH, a tripeptide of glycine, cysteine, and glutamate) which in turn reduces reactive oxygen species (ROS)^15^. However, contradicting the benefit of NAC in treating *Atm*-null mice, increasing oxidative stress by co-deleting either SOD1 or SOD2 (superoxide dismutases), or using different antioxidants (α-tocopherol; Vitamin E), had no impact on survival or tumorigenesis^16^. These latter findings reduced enthusiasm for antioxidant-based treatments of A-T despite apparent benefits of NAC. An alternative scenario is that in the absence of ATM, NAC provides benefit outside of its antioxidant functions.

NAC is a thiol-containing molecule that readily diffuses across the cell membrane^15^. As one of the two sulfur-containing amino acids, intracellular levels of cysteine are tightly regulated; for example, in some cells the import of cystine (dimer of cysteine) is dependent upon the x_c_^−^ antiport system, exchanging cystine for export of a glutamate molecule^17^. x_c_^−^ is a cell surface heterodimer formed by the covalent linkage of two proteins, a heavy and a light chain^18^. The heavy chain, 4F2hc/CD98HC, is the product of the *SLC3A2* gene and is considered to be a cofactor for directing the light chain to the cell surface^19–21^. The light chain is variable and dictates specific amino acid cargos^22^. For instance, in combination with xCT (*SLC7A11*), CD98HC forms the glutamate/cystine antiporter (x_c_^−^), whereas, with y + LAT1/2 (*SLC7A6/SLC7A7*), it forms the cationic/neutral amino acid transporter (y^+^L)^23^.

Here we worked to identify the basis of NAC benefit when ATM function is lost, considering that it may not be through reducing oxidative stress. We discovered that acute cystine and arginine influx concomitant with glutamate efflux is increased following CD98HC phosphorylation by ATM resulting in decreased time for membrane localization of nascent antiporter. Our findings show that NAC treatment rescues a set of A-T phenotypes in cells and mice by rebalancing intracellular glutamate levels in tissues where intracellular glutamate levels depend on x_c_^−^ activity and chronic glutamate accumulation is toxic. We propose a novel mechanistic link where altered regulation of CD98HC-dependent activities in the absence of ATM explains some of the poorly understood clinical manifestations of A-T.

## Results

### ATM activity impacts mitochondrial function and glutamine oxidation in primary human endothelial cells

**Figure 1:**
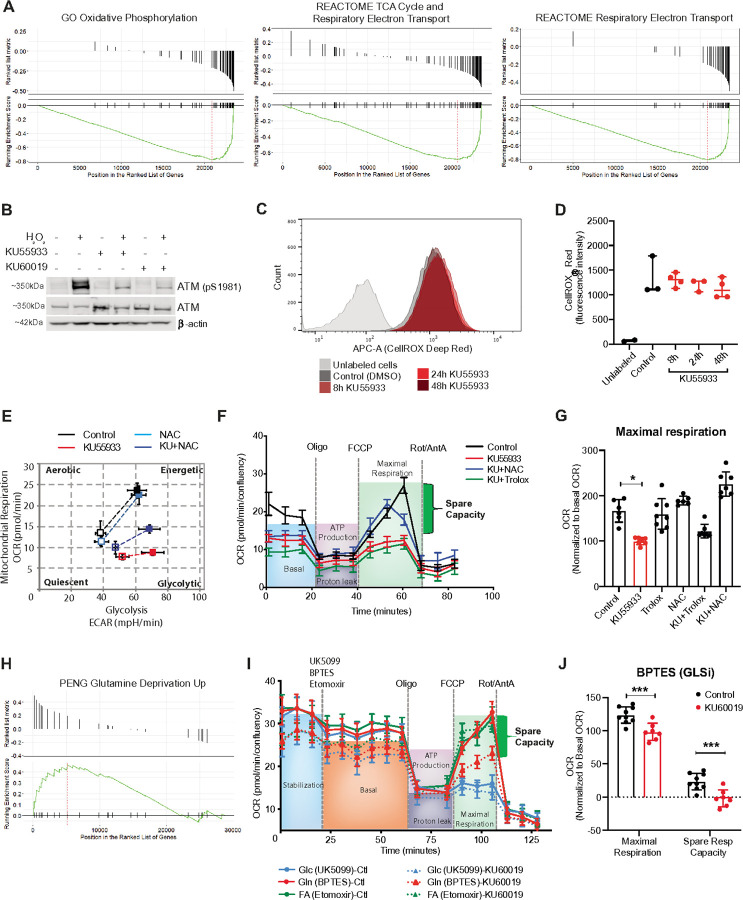
ATMi impacts mitochondrial function and glutamine oxidation in HUVECs. (A) Correlation analysis between expression of ATM and gene sets involved in mitochondrial function from a cohort of normal human tissues (RNA-Seq counts provided by ARCHS^4^ database). (B) Representative blot showing the efficiency of ATMi in HUVECs treated with H_2_O_2_. (C) Flow cytometry chart showing intracellular ROS levels after KU55933 treatment (histogram shows one representative sample per condition). (D) Quantification of ROS levels in C; H_2_O_2_ is used as a positive control. Data represented as median ± 95%CI. (E) Representative graph showing HUVEC’s metabolic potential in response to stressors (Oligomycin and FCCP) after KU55933 and NAC treatments. (F) Representative graph showing mitochondrial function assay after KU55933 treatment, combined with NAC or Trolox. (G) Chart showing maximal respiration obtained in F. (H) Correlation analysis between expression of *ATM* and genes involved in glutamine deprivation (ARCHS^4^ database). (I) Representative graph showing mitochondrial function assay after specific inhibition of glucose, glutamine, or fatty acids oxidation in the presence or absence of ATMi. (J) Chart showing maximal respiration and spare respiratory capacity after inhibition of glutamine oxidation. Data represented as average ± SD. *p<0.05, **p<0.01, ***p<0.001.

ATM is a ubiquitously expressed gene. With the goal of identifying novel physiological roles for ATM, we examined the expression of *ATM* in correlation with other genes across 40,000 normal tissues available from the ARCHS^4^ database using Correlation AnalyzeR^24^. In line with previous reports^9,25,26^, we observed a strong negative correlation between *ATM* and gene expression sets involved in oxidative phosphorylation, citric acid (TCA) cycle, and respiratory electron transport (Fig. 1A; Tables S1–3). To investigate a potential metabolic role for ATM relevant to tissues clinically impacted in A-T but also avoiding concerns of the metabolic impacts often observed with transformed cell lines, we used primary endothelial cells (Human Umbilical Vein Endothelial Cells, HUVEC; relevant to telangiectasia). As ATM is known to be activated by oxidative stress, we carried out this work at tissue-relevant physiological levels of oxygen (3%) and used the well-established pharmacological inhibitors of ATM: KU55933 and KU60019. We confirmed that these ATM inhibitors (ATMi) blocked the H_2_O_2_-mediated activation of ATM (Figs. 1B and S1A) as described with other cell types^27,28^. Under our cell culture conditions, neither ATMi increased intracellular ROS, even after 48 hours (Figs. 1C, 1D, S1B, and S1C); we demonstrated the sensitivity of the ROS assay (CellROX) by treating the cells with increasing doses of H_2_O_2_ (Figure S1D). The same lack of increased basal oxidative stress was recapitulated with shRNA depletion of ATM (Figure S1E) and further confirmed with an alternate method of ROS detection (ROS-Glo^™^ H_2_O_2_ Assay, Figure S1F). Cell proliferation/viability experiments demonstrated that ATMi induced a cytostatic effect after 18 hours (Figures S1G and S1H), recapitulating premature senescence observed with primary A-T or *Atm* null murine fibroblasts^29–32^. To avoid any differences caused by changed proliferative state or viability, we conducted all further investigations with ATMi following 8 hours of treatment.

NAC treatment is known to have some physiological benefits in mice and cells with an ATM defect^13,14^, though other forms of antioxidants failed to provide this benefit. We therefore asked if ATMi impacts any aspect of metabolism in our primary cells that can be rescued by NAC, but not by Trolox (an analog of vitamin E); another potent ROS scavenger. We first measured the oxygen consumption rate (OCR) and the extracellular acidification rate (ECAR) of HUVECs, with and without ATMi, as indicators of mitochondrial respiration and glycolysis, respectively. Sequential injections to determine the glycolytic flux after ATMi did not show a significant change (Figure S1I). However, upon injection of stress inducers (Oligomycin and FCCP), HUVECs displayed a strong shift towards glycolysis with ATMi rather than a balanced upregulation of both aerobic respiration and glycolysis as observed in the control (Fig. 1E); this effect was partially rescued by NAC treatment. Examining the OCR results in more detail, we observed a strong impairment in basal respiration, maximal respiration, and spare respiratory capacity following ATMi (Figs. 1F and S1J). The impairment of basal OCR was not altered by NAC or Trolox treatment. In contrast, NAC, but not Trolox, rescued the maximal respiration and spare respiratory capacity defects (Figs. 1G and S2A) indicating an impact on mitochondrial metabolism. Since we were interested in identifying a NAC-specific effect, these observations became the focus of our studies.

Given that ATMi reduces the ability to upregulate aerobic respiration, we considered the possibility that this may be the result of altered TCA cycle and electron transport chain (ETC) use. Supporting this concept, examining the ARCH^4^ co-expression analysis further revealed a positive correlation between ATM expression and genes upregulated in response to glutamine deprivation (Fig. 1H; Table S4). Consequently, we measured the impact of ATMi on mitochondrial fuel oxidation using specific inhibitors of glucose, glutamine, and fatty acids oxidation (UK5099, BPTES, and etomoxir, respectively). Blocking glucose oxidation clearly impaired mitochondrial function irrespective of treatments (Figs. 1I and S2B left panel); this was expected since endothelial cells are highly glycolytic^33^. No effect was observed after blocking fatty acids oxidation (Fig. 1I and S2B right panel). Strikingly, blocking glutamine oxidation caused mitochondrial dysfunction only with ATMi indicating a greater dependency upon glutamine (Figs. 1J and S2C).

### Inhibition of ATM alters glycolysis and the pentose phosphate pathway (PPP) activity

**Figure 2:**
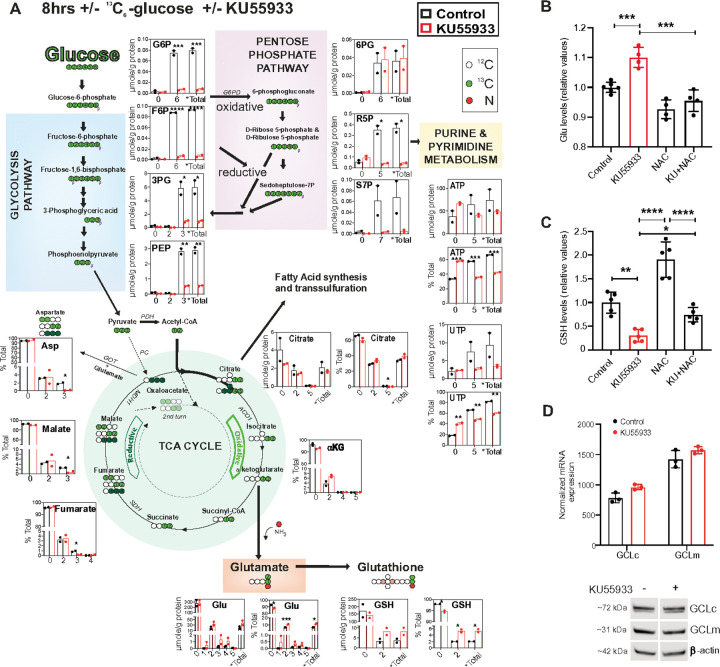
ATMi rewires metabolism leading to glutamate accumulation and glutathione depletion. (A) Schematic of the ^13^C_6_-glucose tracing showing the major significant changes observed in glycolysis and TCA cycle after ATMi in HUVECs. (B-C) Charts showing intracellular glutamate and GSH levels in cells treated with KU55933, NAC, and in combination. (D) Chart showing mRNA levels and blots showing protein levels of GCLc and GCLm after ATMi. Data represented as average ± SD. *p<0.05, ***p<0.001, ****p<0.0001.

To further characterize the metabolic changes induced by ATMi, we performed a stable isotope resolved metabolomics (SIRM) study using [U-^13^C]-labeled glucose. In line with previous reports^11,34^, we observed reduced glycolysis and PPP metabolites after ATMi treatment based on ^13^C incorporation (Fig. 2A blue and pink panels); this can indicate either a block in these pathways preventing production of metabolites or increased flux depleting the observed metabolites. For PPP, a clear block in the pathway was noted in the 6PG dehydrogenase step; compare unaltered 6PG ^13^C labeling pattern to reduced labeling in R5P after ATMi. To confirm this block, we measured NADPH levels by measuring the NADP^+^/NADPH ratio (Figure S2D). For glycolysis, despite a clear reduction of incorporated label upon ATMi evident from glucose-6-phosphate (G6P) onwards, no apparent accrual of any metabolite was observed. This result suggests increased glycolysis which was also indicated by the Seahorse assays (Fig. 1E), which, if correct, would result in increased flux into the Krebs/TCA cycle. Of note, ATMi did not significantly alter amount of glucose derived lactate in the media (*data not shown*).

### ATM inhibition alters TCA cycle while accumulating glutamate

To assess the impact of ATMi on Krebs cycle we tracked ^13^C label from glycolysis into the Krebs cycle (Fig. 2A green panel) and noted an overall decrease in glucose-derived ^13^C upon ATMi, ~ 5% for most metabolites, other than citrate which was reduced about 50%. We also noted the loss of the pyruvate carboxylase (PC; dark green ^13^C) activity following to ATMi as shown by a significant decrease in “reductive” ^13^C_3_-malate and ^13^C_3_-fumarate, as well as ^13^C_3_-Asp production and “oxidative” ^13^C_5_-citrate compared to control cells. In contrast, the “oxidative” production of ^13^C_2_-glutamate (Glu) (Fig. 2A) via citrate to a-ketoglutarate was increased upon ATMi treatment while a lesser increase is seen with percent ^13^C_2_-glutathione (GSH). These results suggest increased glucose flux through glycolysis and TCA cycle to glutamate. This increased label was confirmed by NMR analysis (Figure S2E and F). However, though ^13^C increased in both Glu and GSH, the total amount (unlabeled + labeled) of Glu increased (299 to 327 μmoles/g) while GSH decreased (176 to 150 μmoles/g). These data firstly indicate that in HUVEC cells the majority of the Krebs cycle metabolites are likely derived from non-glucose source(s) such as glutamine. Secondly, they indicate that ATMi results in increased glucose-derived ^13^C flowing through oxidative TCA cycle to Glu and GSH. To better understand these observations, we performed an analogous SIRM experiment with [U-^13^C,^15^N]-glutamine (Figure S2G) and found that glutamine indeed contributes significantly to the pool of Krebs cycle metabolites irrespective of ATMi (total ^13^C enrichment; Figure S2G *Total). Key points noted from this experiment were (i) oxidative TCA cycle, the main mechanism for ^13^C-citrate production in HUVEC cells, was increased while reductive carboxylation was decreased by ATMi and (ii) that ATMi treatment resulted in more *de novo* GSH production without increasing overall GSH levels. In contrast, following ATMi, it appeared that both unlabeled and labeled glutamate levels increased, with an increased proportion of labeled glutamate, including use of aKG as a source (indicated by either ^13^C and no ^15^N, or ^15^N and no ^13^C). Overall, these data corroborate the Seahorse results, with ATMi impacting basal TCA cycle function. They also support the ^13^C-glucose data, with ATMi causing an increase in glutamate levels derived from both glucose derived aKG and glutamine, but with some impairment in GSH production despite some labeled *de novo* GSH. To further confirm the effect of ATMi on glutamate levels, we directly assessed glutamate levels and found that ATMi does in fact induce a significant increase (~ 10%) in intracellular glutamate over the 8h of ATMi exposure (Fig. 2B). To independently confirm this observation, we examined primary *Atm*^*−/−*^ mouse embryonic fibroblasts (MEFs) and found that these constitutively null cells display a chronic accumulation of intracellular glutamate compared to controls (Figure S2H).

Having observed that ATMi causes glutamate accumulation in both SIRM experiments as well as increased labeled GSH without increasing total amounts of GSH, we decided to investigate the impact of ATMi on GSH levels more directly. We found that GSH levels decreased significantly in HUVEC upon ATMi treatment (Fig. 2C). Given the prior benefits we observed for NAC in our Seahorse assays, we tested if NAC could restore intracellular GSH, which it did (Fig. 2C). NAC relieves the rate-limiting component of GSH production by providing excess cysteine, allowing the production of the tripeptide when combined with glycine and glutamate. Considering this, we examined the effect of NAC on ATMi-increased glutamate levels and noted a significant reduction (Fig. 2B). Based on this data and the ^13^C_5_,^15^N_2_-glutamine-based SIRM experiment showing that ATMi induces a depletion of unlabeled GSH though still accumulating ^13^C-GSH (Figure S2G) we reasoned that both GSH utilization and synthesis increased in response to ATMi treatment, though without apparent accumulation of ROS and without the ability to restore normal GSH levels. Of note, we observed no decrease in the expression of enzymes used in the production of GSH, GCLc/GCLm (Fig. 2D). Given no apparent defect in the enzymes necessary for GSH production, we considered that the production defect may result from limited substrate availability, most notably cysteine, given that this is the rate limiting component and would be rescued by NAC treatment.

### ATM phosphorylates CD98HC to regulate cystine uptake and glutamate export

**Figure 3:**
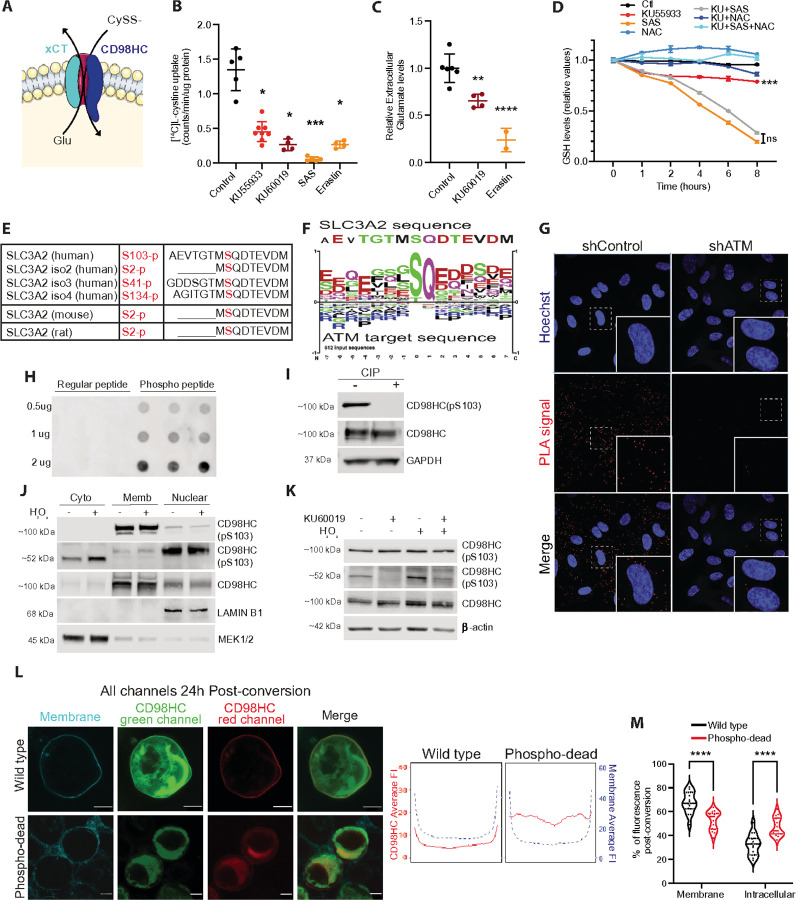
ATM modulates the xc− antiport system through phosphorylation of CD98HC. (A) Representation of the x_c_^−^ antiport involved in cystine/glutamate transport (created using SMART; www.smart.servier.com). (B) ^14^C-L-cystine uptake after treatment with KU55933 or KU60019; SAS and Erastin are used as specific inhibitors of x_c_^−^. (C) Fluorometric analysis of extracellular levels of glutamate after ATMi. (D) Chart showing GSH levels measured at different times following ATMi, x_c_^−^ inhibition (SAS) or the combination (n=2). Data represented as average ± SD. *p<0.05, **p<0.01, ***p<0.001, ****p<0.0001. (E) Table showing the highly conserved SQ site in SLC3A2. (F) ATM consensus motif (www.phosphosite.org)^69^. (G) Representative images of proximity ligation assay (PLA) showing the interaction between CD98HC and ATM, shRNA-ATM cells were used to show signal specificity (Magnification: 80X and 200X for insets). (H) Dot blot showing binding specificity of the newly synthesized phospho-antibody. (I) Blots to further confirm antibody specificity in cell lysates treated with alkaline phosphatase (n=2). (J) Subcellular fractionation after 2h of treatment with H_2_O_2_. MEK1/2 and LAMIN B1 were used as cytoplasmic and nuclear markers, respectively._-_(K) Blots showing the detection of phosphorylated CD98HC, its induction by H_2_O_2_, and inhibition by KU60019. (L) Representative images of the photoconversion assay in HEK293 cells transfected with SLC3A2 wild type or phospho-dead (scale bar: 5μm). The line plots on the right show averaged fluorescence intensity from several cell cross-section profiles (also see Figure S4B). (M) Chart showing quantification of fluorescence intensity 24h after photoconversion (n=15, from two independent experiments).

In response to ATMi or in ATM knockout MEFs, we noted an increase in intracellular glutamate levels and decreased GSH levels and that these defects were counteracted by NAC; these results indicate a defect in cysteine availability. Intracellular levels of cysteine are tightly controlled, being either produced *de novo* from methionine and serine or, more often, actively imported from outside the cell; a process bypassed by NAC. We, therefore, performed RNA-seq to identify any gene expression changes upon ATMi treatment and noted upregulation of genes involved in *de novo* cysteine synthesis from serine and methionine (Figure S3A), suggesting a compensatory response to maintain intracellular cysteine levels. This led us to hypothesize that ATM could be involved in promoting the activity of the cystine/glutamate antiport system, x_c_^−^ (Fig. 3A). Using radiolabeled cystine, we found that cystine import was significantly impaired by ATMi (Fig. 3B) or shATM knockdown (Figures S3B and S3C). Consistent with a role for ATM in stimulating basal x_c_^−^ activity we also found that the level of extracellular glutamate was lower following ATMi compared to untreated control (Fig. 3C). Sulfasalazine (SAS) and Erastin, two specific inhibitors of the x_c_^−^ antiport system, were used as controls for the experiments and displayed a more profound effect than ATMi. Overall, inhibition of ATM function results in a partial decrease in x_c_^−^ activity, leading to an accumulation of intracellular glutamate and insufficient cystine import to maintain basal levels of GSH in the cell.

Considering that we observed no increase in ROS following ATMi for HUVECs grown at 3% O_2_, we decided to evaluate the reduced/oxidized state of GSH/GSSG. We observed a decrease in the ratio of GSH/GSSG following 8 hours of ATMi treatment, but the change in absolute GSSG levels was not significant (Figure S3D). This result supports the concept that there is no inherent increase of ROS upon ATMi when cells are maintained at 3% O_2_, but there is a defect in maintaining GSH levels. This goes in line with the decreased NADPH production due to decreased oxidative PPP activity, as NADPH is used by glutathione reductase to recycle GSH from GSSG. To better understand how ATM impacts intracellular levels of GSH pools we conducted a time course experiment to monitor GSH changes (Fig. 3D). Interestingly, ATMi alone caused a significant initial decrease (in 1–2 hours) of intracellular GSH levels, which then stabilized without returning to control levels in the 8 hours monitored. This observation is consistent with our conclusion that the increase in *de novo* synthesis of cysteine is an attempt to compensate for decreased cystine import. As expected, complete inhibition of x_c_^−^ by SAS caused a dramatic depletion of GSH. NAC treatment rescued normal GSH levels irrespective of SAS or combined SAS and ATMi. From this data, it appears that ATM augments x_c_^−^ antiport activity under basal conditions. However, ATM is best understood for activating a pleiotropic cellular response to damage, particularly to ionizing radiation. To explore this possibility, we used primary MEFs and noted that though ionizing radiation induced a strong influx of cystine irrespective of ATM status, the amount of uptake by *Atm*^*−/−*^ MEFs was significantly less than that observed for *Atm*^*+/+*^ MEFs (Figure S3E). Again, these data support the notion that ATM augments x_c_^−^ antiport activity, in this case in response to ionizing radiation.

Given the concept that ATM regulates x_c_^−^ antiport activity, the most likely mechanism would involve a direct phosphorylation event by the ATM kinase. Evaluating the sequences of the two proteins that constitute x_c_^−^ antiport revealed that CD98HC has a highly conserved SQ site in its intracellular tail (S103, Fig. 3E) that is embedded within a canonical ATM target motif (Fig. 3F). To determine if CD98HC is a substrate of ATM, we first demonstrated their interaction using the proximity ligation assay (PLA, Figs. 3G and S3F) and confirmed it by co-immunoprecipitation (Figure S3G). We then generated a phospho-specific monoclonal antibody against CD98HC (S103, Figs. 3H and 3I); this antibody detected the phosphorylation in both glycosylated CD98HC (~ 100kDa) and native CD98HC (~ 50–60kDa, Fig. 3J). Treatment with the protein glycosylation inhibitor tunicamycin verified reduction of the ~ 100kDa glycosylated CD98HC with concomitant increase in ~ 50–60kDa unglycosylated CD98HC (Fig S3H). Detection of phosphorylation of native CD98HC indicates that ATM phosphorylates this protein before it covalently binds to its partner protein and localizes to the cell membrane. Subcellular protein fractionation experiments confirmed that CD98HC phosphorylation at S103 occurs in the cytoplasm (Fig. 3J). CD98HC phosphorylation was increased upon induction of ATM by H_2_O_2_, but both basal and induced phosphorylation was abolished by ATMi (Figs. 3K and S3I). To confirm that no other DNA damage responsive PIKK family member phosphorylates CD98HC S103, at least in response to H_2_O_2_, we used inhibitors of DNA-PKcs and ATR (AZD7648 and AZD6738, respectively) and observed no impairment (Figure S3J). Given that AKT can sometimes mediate an indirect ATM phosphorylation event we also inhibited AKT using MK2206, but again observed no impairment of CD98HC phosphorylation (Figure S3K).

CD98HC is understood to act like a chaperone, facilitating antiport localization to the cell surface. We therefore developed an assay to evaluate the rate of CD98HC trafficking to the membrane by fusing CD98 to the photoconvertible protein mEos3.2. Upon photoconversion all mEos3.2 present in the cell shifts from green to red fluorescence while any subsequently produced mEos3.2 will remain green fluorescent. We can then monitor where and what proportion of the red mEos3.2 locates at specific times. With this assay we demonstrated a clear impairment of the S103A mutant (phospho-dead) CD98HC::mEos3.2 trafficking to the membrane compared to wildtype sequences (Fig. 3L, 3M and S4A, S4B). Overall, our findings demonstrate that ATM interacts with and phosphorylates the intracellular 52 kDa monomeric CD98HC in response to oxidative stress to increase the rate of *de novo* antiport trafficking to the cell surface, providing a mechanistic basis for the impaired cystine import and glutamate accumulation observed with ATM inhibition.

### ATM phosphorylation is required for optimal angiogenesis

**Figure 4:**
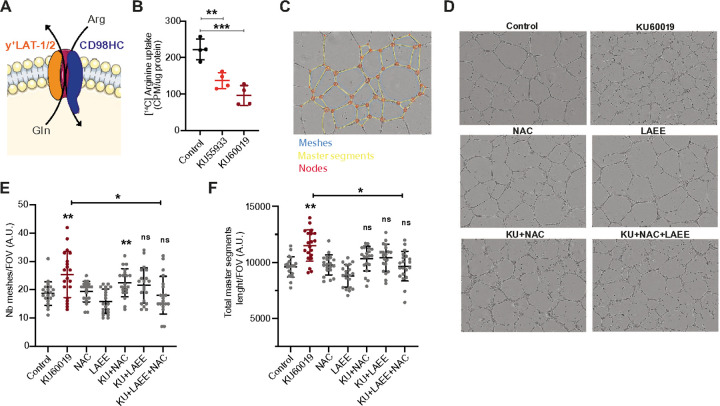
ATM phosphorylation of CD98HC impacts angiogenesis. (A) Drawing showing one of the two antiport systems involved in arginine uptake in endothelial cells (created using SMART). (B) Representative chart showing ^14^C-L-arginine uptake after ATMi. (C) Picture showing the parameters evaluated in the vessel formation assay. (D) Representative images of the angiogenesis assay. (E-F) Quantification of images using the Angiogenesis Analyzer from Image J. Data represented as average ± SD. *p<0.05, **p<0.01, ***p<0.001.

Given that ATM-dependent phosphorylation increases x_c_^−^ antiporter activity by increasing its cytoplasm to membrane localization, we asked two key questions: does the impact of ATM phosphorylation of CD98HC extend to other antiporters? Does reduced inducible antiporter activity in response to ATMi have any physiological impact? To address these questions, we considered another antiport system that involves CD98HC, the y^+^L system. This antiporter is formed between CD98HC and y^+^LAT1/2 (SLC7A6/SLC7A7) and has high affinity for the uptake of arginine^35–37^ (Fig. 4A). Arginine import is essential for nitric oxide (NO) synthesis and angiogenesis^38,39^; a function pertinent to the telangiectasia phenotype of A-T. We found that ATMi (Fig. 4B) or shATM depletion (Figure S4C) significantly reduced [^14^C]-L-arginine uptake. Given this observation we went on to test the impact of ATMi on endothelial vessel formation and noted a clear impairment (Figs. 4C and 4D). The network of capillaries formed in the presence of ATMi had a higher number of meshes and master segments length compared to the control (Figs. 4E and 4F) resulting in a shorter mesh index (Figure S4D). These phenotypes indicate an inability of ATM-inhibited HUVECs to migrate and form the same lumen width observed for control cells. Supporting our model that inhibiting ATM decreases both x_c_^−^ and y^+^L activity, we found that combining NAC and L-Arginine Ethyl Ester (LAEE) (cell-permeable forms of cysteine and arginine, respectively) restored normal angiogenesis irrespective of ATMi. Interestingly, using the wound healing scratch assay, we found that ATMi impaired migration which was restored upon NAC treatment (Figures S4E and S4F). These results are further supported by gene expression analysis showing that ATMi modulates the expression of genes involved in migration and vessel formation in endothelial cells (Figure S4G). Overall, these results demonstrate that ATM is involved in angiogenesis by modulating the activity of both x_c_^−^ and y^+^L transporters and that their decreased activity upon ATMi can be bypassed by the addition of both NAC and LAEE.

### ATM phosphorylation of CD98HC impacts alpha and beta pancreatic cells

**Figure 5:**
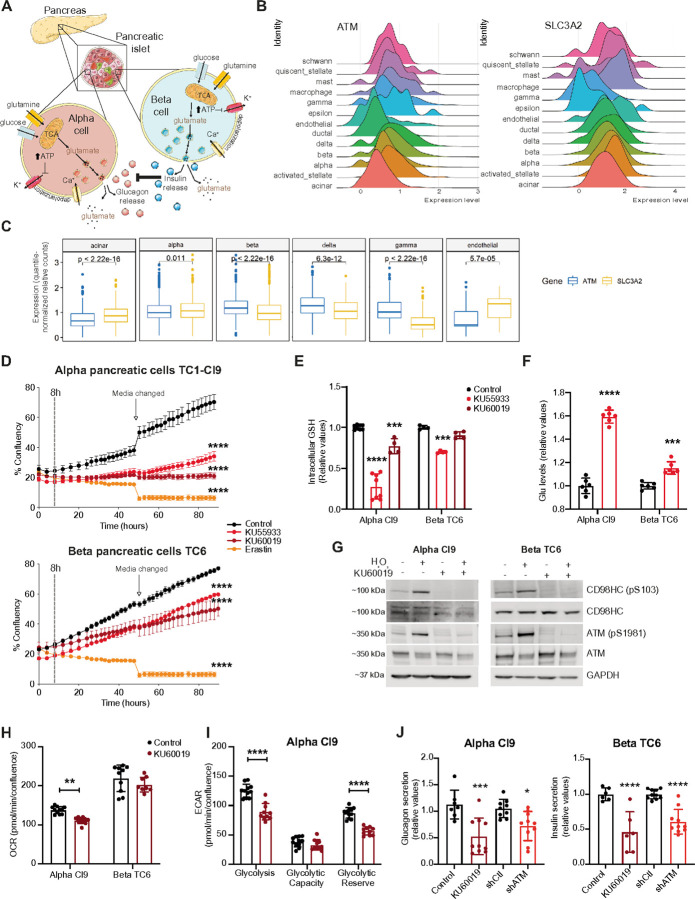
Pancreatic a and b cells are highly sensitive to ATM and x_c_^−^-inhibition. (A) Simplified illustration showing TCA cycle and ATP levels modulating insulin and glucagon release (created using SMART). (B) Ridge plots showing *ATM* and *SLC3A2* heterogeneous expression in human pancreatic cells (data obtained from multiple scRNA-Seq studies). (C) Box plots showing quantile-normalized *ATM* and *SLC3A2* expression in different human pancreatic cell types. (D) Representative confluency charts of a and b cells following ATMi and x_c_^−^ inhibition (Erastin). (E-F) Intracellular GSH and glutamate levels after ATMi in a and b cells. (G) Representative blots showing CD98HC phosphorylation (S103) after H_2_O_2_ and ATMi in a and b cells. (H-I) Basal respiration of a and b cells and glycolytic function of a cells after ATMi. (J) Glucagon and insulin secretion in a and b cells after ATMi/knockout. Data represented as average ± SD. *p<0.05, **p<0.01, ***p<0.001, ****p<0.0001.

Having established an acute effect of CD98HC phosphorylation on the activity of associated antiporters, we wanted to determine whether this consequence relates to a progressive phenotype pertinent to A-T, a chronic disease. A-T patients are known to develop abnormalities in glucose homeostasis with 25% of those patients who survive to age 30 developing diabetes mellitus^40,41^. Similar to neurons, pancreatic α and β cells use glutamate as a signaling molecule^42,43^ and are highly sensitive to glutamate accumulation^44^ (Fig. 5A). Thus, we considered the possibility that loss of ATM could lead to a chronic accumulation of glutamate in pancreatic islets with a potential toxic impact. To explore this possibility, we first evaluated single-cell RNA sequencing data from multiple studies of the human pancreas (GSE81076, GSE85241, GSE86469, E-MTAB-5061) and noted heterogeneity in *ATM* expression across different cell types (Figs. 5B and S5A). Expanding on this, we observed an inverse relationship between *ATM* and *SLC3A2* expression levels (Fig. 5C); indicating that, in this tissue, the absence of ATM-induced activity is compensated by the increased presence of the antiporter. To explore this relationship further we turned to use murine αTC1 Clone 9 and β-TC6 pancreatic cells as an *in vitro* model. Both cell types were sensitive to ATMi (Fig. 5D), though the effect was clearly stronger in α cells. In addition, both cell types were extremely sensitive to erastin, substantiating their reliance on the x_c_^−^ antiport system. We did note that α cells showed a non-significant increase in ROS levels after ATMi that we did not observe in β cells (Figures S5B and S5C). Similar to HUVECs, ATMi caused a decrease in intracellular GSH levels and a concomitant increase in intracellular glutamate in both α and β pancreatic cells (Figs. 5E and 5F). Of note, the relative increase in intracellular glutamate in α cells induced by ATMi is much higher than that observed in β cells though the absolute basal glutamate levels are higher in β cells compared to α cells (Figure S5D). If glutamate toxicity is the basis of ATMi induced death we would expect that its clearance by NAC facilitated GSH production and rescue viability, while ester-GSH (eGSH) would not confer this benefit despite reducing ROS levels, which is what we observe (Figure S5E and S5F). Comparable with HUVECs, H_2_O_2_ treatment induced CD98HC phosphorylation in an ATM-dependent manner in both α and β cells (Fig. 5G). We then asked if islet cells demonstrated an ATM-dependent metabolic change similar to that observed with HUVECs. Here, we found a significant impact on mitochondrial respiration and glycolysis following ATMi in α cells (Figs. 5H and 5I). In contrast, the decrease observed with β cells was milder and not significant (Figs. 5H and S5G). Finally, considering the functional role of pancreatic islet cells, we asked whether ATMi or depletion impacts hormone secretion and found that indeed glucagon and insulin secretions were impaired in α and β cells respectively (Fig. 5J). Of note, glutamine derived aKG followed by reductive TCA cycle has been shown to be required for insulin secretion^45^, which reconciles well with the impacts on metabolism and impaired insulin secretion we observe with ATMi. Overall, our findings indicate that pancreatic islet cells, particularly α cells, are impacted by the loss of ATM function, affecting cell viability, glutamate accumulation, GSH production in response to oxidative stress, and hormone secretion.

### Atm deficiency results in glucose intolerance and pancreatic islets malfunction

**Figure 6:**
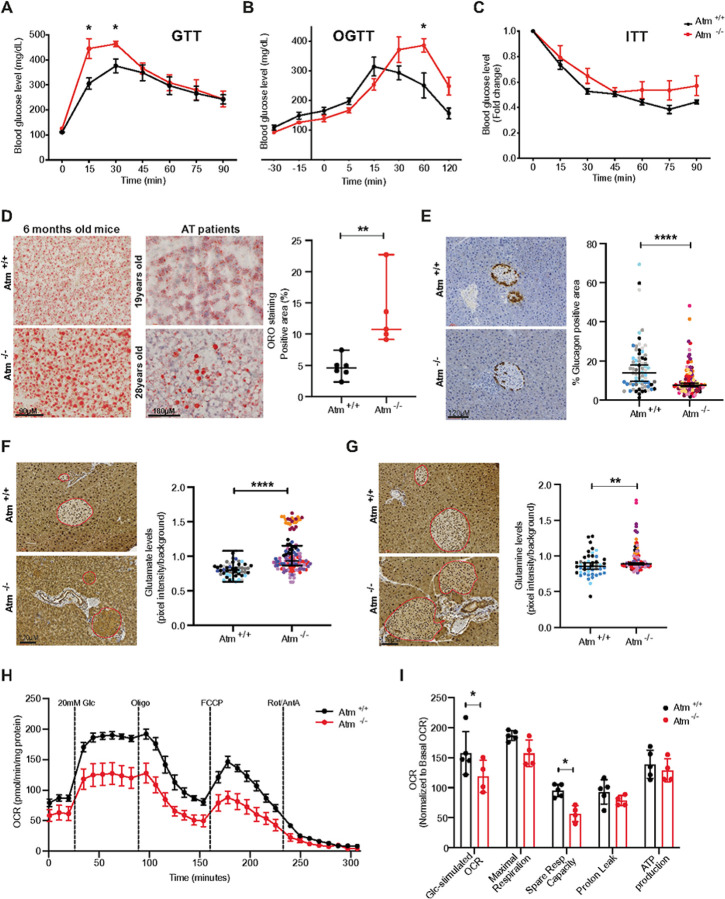
ATM deficiency impairs pancreatic islet function leading to glucose intolerance and hepatic lipid accumulation. (A-B) Intraperitoneal and oral glucose tolerance test in *Atm*^*+/+*^ and *Atm*^*−/−*^ mice (n=4–6). (C) Glucose measurements after intraperitoneal injection of insulin (ITT, n=4–6). Data represented as average ± SEM. (D) Representative images and quantification of lipid levels by Oil Red O staining in the liver from 6-month-old *Atm*^*+/+*^ and *Atm*^*−/−*^ mice (n=5–6) and A-T patients. Data represented as average ± SD. (E) Representative images of glucagon staining in pancreas from *Atm*^*+/+*^ and *Atm*^*−/−*^ mice and resultant quantification (each dot represents a single islet, n=5–8). (F-G) Representative images and quantification of glutamate and glutamine staining in pancreatic islets of *Atm*^*+/+*^ and *Atm*^−/−^ mice (each dot represents a single islet, n=5–8). Data represented as median ± 95%CI. (H) Mitochondrial respiration of pancreatic islets isolated from 6-month-old *Atm*^*+/+*^ and *Atm*^−/−^ mice (n=4–5). (I) Parameters evaluated in H show glucose response and mitochondrial performance in *Atm*^*+/+*^ vs. *Atm*^*−/−*^ mice (each dot represents an animal). Data represented as average ± SEM. *p<0.05, **p<0.01, ****p<0.0001.

Our observation that loss of ATM activity impacts α and β cell function in culture offered a tractable model to test *in vivo*. Others reported an insulin secretion defect in *Atm*^*−/−*^ mice^46^; however, the underlying mechanism was not elucidated. We therefore set out to characterize glucose homeostasis of *Atm*^−/−^ mice fed with a standard chow diet. Using the glucose tolerance test we found that *Atm*^−/−^ male mice developed glucose intolerance by 6 months of age while female mice displayed this defect by 1 year (Figs. 6A, 6B and S6A). In contrast, insulin sensitivity assessed with an insulin tolerance test was similar between genotypes (Fig. 6C). To further study these responses, we examined the impact of fasting or feeding. When fasted, we observed no difference between the genotypes, but when allowed to feed, blood levels of insulin were significantly lower in *Atm* ^*−/−*^ mice while glucose levels increased (Figures S6B and S6C). Based on our cell culture work, we expected that the absence of ATM would impact α cell function, particularly by reducing the amount of glucagon, inducing fatty acid utilization in the liver. Substantiating our cell-based findings we found that *Atm* ^−/−^ mice develop fatty liver (Fig. 6D), which may be related to lower hepatic fat oxidation from the reduced glucagon. In fact, we observed the same increased accumulation of lipids in liver samples from A-T patients; this finding matches a clinical report using a larger cohort of A-T patients^47^. To further understand the impact of ATM deficiency on α cells, we evaluated the percentage of glucagon-expressing cells (indicating the presence of α cells) in the pancreatic islets of 6-month-old mice. We observed a significant reduction in the percent of islet area that was glucagon positive in *Atm* ^*−/−*^ compared to *Atm* ^*+/+*^ mice (Figs. 6E and S6D). We found no differences in pancreatic islet size or CD98HC expression between genotypes (Figures S6E and S6F). Strikingly, and in line with our cell culture findings, pancreatic islets of 6-month-old *Atm* ^*−/−*^ mice accumulated higher levels of glutamate and glutamine compared to *Atm* ^*+/+*^ mice (Figs. 6F, 6G and S6G). To confirm our findings we performed the same analysis using an independent *Atm* null model (*Atm*^*tm1Fwa*^, 3–5 month-old)^48^ and observed similar results, increased glutamate and glutamine levels in the *Atm* null pancreatic islets compared to wildtype (Figure S6H). Finally, having observed a strong recapitulation of our cell culture results we went on to determine if we would also observe the same metabolic defect in islets. For this, we used an *ex vivo* system, isolating pancreatic islets from *Atm*^*+/+*^ and *Atm*^*−/−*^ mice. We found glucose response was significantly impaired in islets isolated from *Atm* ^*−/−*^ compared to *Atm* ^*+/+*^ mice (Figs. 6H and 6I). Mimicking our cell culture results, *Atm*^*−/−*^ islets showed a similar impairment in the spare respiratory capacity as well as a lower level of basal respiration (Figs. 6I and S6I). Altogether our findings demonstrate that the absence of ATM results in a progressive chronic metabolic defect in pancreatic islets with loss of α cell viability and an eventual decline of endocrine functions.

### N-Acetyl Cysteine rescues glucose intolerance, glutamate accumulation and glucagon production in the pancreatic islets of ATM-deficient mice.

**Figure 7:**
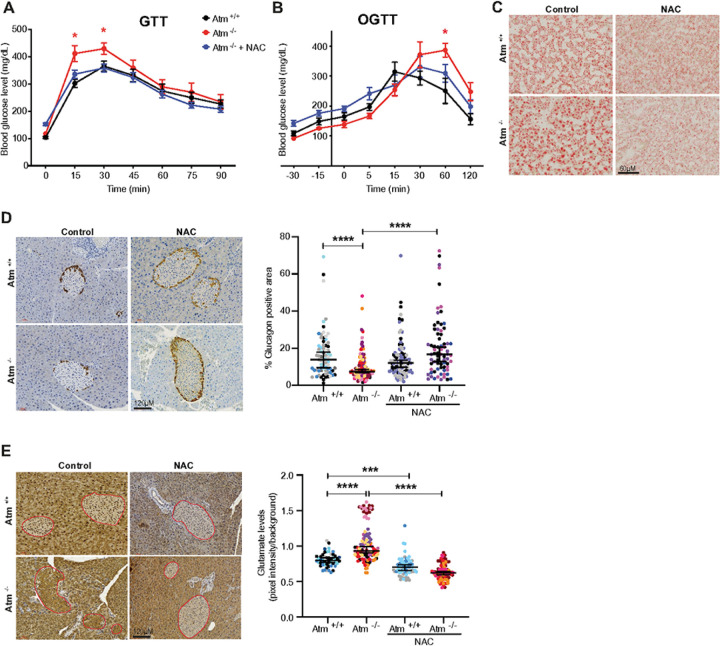
NAC supplementation rescues the metabolic defects shown in ATM-deficient mice. (A-B) Intraperitoneal and oral glucose tolerance test in *Atm*^*+/+*^ and *Atm*^*−/−*^ mice supplemented with NAC (n=4–8). Data represented as average ± SEM. (C) Representative images of Oil Red O staining in the liver from 6-month-old *Atm*^*+/+*^ and *Atm*^−/−^ mice supplemented with NAC. (D) Representative images of glucagon staining in pancreas from *Atm*^*+/+*^ and *Atm*^−/−^ mice and resultant quantification (each dot represents a single islet, n=3–8). (E) Representative images and quantification of glutamate staining in pancreatic islets of *Atm*^*+/+*^ and *Atm*^−/−^ mice (each dot represents a single islet, n=3–8). Data represented as median ± 95%CI. *p<0.05, ***p<0.001, ****p<0.0001.

Our findings in cells and mice led to the prediction that the underlying consequence of a lack of ATM activity in the pancreas is an accumulation of toxic levels of glutamate through an inability to acutely regulate cysteine levels. If this model is correct, then NAC supplementation should allow GSH production and thereby alleviate toxic glutamate accumulation and prevent the glucose intolerance observed in *Atm*^*−/−*^ mice. We, therefore, supplemented the drinking water of our mice with NAC from conception throughout their life. NAC treatment rescued both the glucose intolerance and hepatic lipid accumulation observed in *Atm* ^*−/−*^ mice (Figs. 7A, 7B and 7C). Interestingly, we went on to show that NAC treatment of *Atm*^*−/−*^ mice rescued the percentage of glucagon-positive areas in the pancreatic islets and reduced glutamate accumulation to normal levels (Figs. 7D, 7E, S6J and S6K). These results clearly show that in the absence of ATM, NAC supplementation works to reduce the chronic accumulation of toxic levels of intracellular glutamate pools in pancreatic islets thereby allowing maintenance of glucose homeostasis.

## Discussion

In this study, we aimed to find novel ATM targets/functions that improve our mechanistic understanding of A-T phenotypes and may lead to new treatments for these patients. We started by characterizing the metabolic function of ATM in primary endothelial cells (HUVECs), relevant to the telangiectasia phenotype of A-T. In line with previous reports using A-T fibroblasts, thymoblasts, and cardiomyoblasts^9,26^, we observed impairment in mitochondrial function of HUVECs after ATMi. However, we did not see an increase in ROS levels upon ATMi as was reported before with ATM-deficient cells^25,49^; this is most likely due to the lower oxygen levels for our cell cultures (3% O_2_) as opposed to 21% in other studies which is expected to increase oxidative stress levels^50^. Despite ATMi not increasing oxidative stress in our models, we found HUVECs to be very sensitive to ATMi, suggesting an essential ROS independent role for ATM in basal maintenance of endothelial cells; under basal conditions, activated ATM is present in the cytoplasm (Figure S1A).

Following these observations, we employed [U-^13^C]-glucose and [U-^13^C, ^15^N]-glutamine in stable isotope tracing to better understand metabolic reprogramming in HUVEC induced by ATMi. We found compromised PPP and an altered glycolysis and mitochondrial anaplerosis that resulted in increased glucose derived label present in glutamate and increased accumulation of glutamate following ATMi; critically this accumulation was rescued by NAC treatment. We went on to find that despite glutamate accumulation, total GSH levels were reduced after ATMi. With no apparent defect in the expression of the enzymes used for GSH production, we went on to examine substrate availability. Given that cysteine is the rate-limiting component for GSH production and NAC rescues A-T phenotypes, we pursued the potential role for ATM in controlling intracellular cysteine levels.

We first demonstrated a cystine uptake defect in ATMi-HUVECs as well as a decrease in extracellular export of glutamate compared to control cells. A similar effect was observed with *Atm*^*−/−*^ primary MEFs which were not able to invoke as strong a cystine uptake response following irradiation as wild-type cells, supporting our findings with the pharmaceutical inhibitors. Next, we showed that the interaction of ATM and CD98HC led to the identification of a highly conserved ATM target phosphorylation site in the intracellular tail of CD98HC (S103). After developing a phospho-specific antibody, we showed that H_2_O_2_-induced monomeric CD98HC S103 phosphorylation was ATM dependent (Fig. 3K). Interestingly, we did note that the phospho-dead CD98HC had a reduced rate of CD98HC trafficking to the cell membrane (Fig. 3L and 3M). These data provide mechanistic insight into the functional consequence of this phosphorylation that fits with the observed phenotypes.

Our finding that ATM phosphorylates CD98HC is the first demonstration that posttranslational modification of CD98HC can impact overall antiport channel activity, albeit by increasing the rate of trafficking to the cell membrane. Considering this, we examined the y^+^L channel, a heterodimer of y^+^LAT1/2 and CD98HC, an antiporter used by endothelial cells to import arginine^23^. We found that either ATMi or ATM depletion significantly reduced arginine uptake which corresponded to an angiogenesis defect. In line with our data, Jia et al. showed that ATM haploinsufficiency reduced angiogenesis after myocardial infarction in mice^51^. Endothelial dysfunction is a hallmark of atherosclerosis progression and blood vessel function^52,53^. Concordantly, our findings provide new insights in regards to the exacerbated atherosclerosis observed in *Atm*^*−/−*^*ApoE*^*−/−*^ mice models and the ocular telangiectasia of A-T patients^54,55^.

One poorly characterized phenotype of A-T patients is diabetes^41^. Since α and β pancreatic cells respond to glutamate levels and metabolism of this amino acid by reductive TCA cycle secrete glucagon and insulin^44,45,56^, we considered that the accumulation of intracellular glutamate upon loss of ATM function may impact these cells. We were able to recapitulate the ATMi induced phenotypes we observed in HUVECs in immortalized α and β cells, with the additional consequence of impaired hormone secretion. These results indicate that ATM modulation of x_c_^−^ antiport activity impacts pancreatic cells performance. To demonstrate this premise, we used *Atm*^*−/−*^ mice^31,57^. Similar to another *Afm*-deficient mouse model^46^, our *Atm*^*−/−*^ mice developed glucose intolerance, most likely related to the reduction in postprandial insulin secretion. In agreement with an insulin secretion defect, the *Atm*^*−/−*^ mice developed fatty livers, a condition characteristic of type 2 diabetes and reported in A-T patients^47,58^. We also observed a reduced amount of glucagon-producing α cells in *Atm*^*−/−*^ mice compared to *Atm*^*+/+*^; this loss of α cells provides an explanation for the observed fatty liver phenotype. Overall, both our animal- and cell-based work demonstrate that without functional ATM there is a defect in endocrine activity in pancreatic islets. As we were not able to observe any apoptotic cells in *Atm*^*−/−*^ pancreatic islets by TUNEL assay (*data not shown*), we surmise that the underlying defect was due to the release of glucagon and insulin by the α/β pancreatic cells respectively. Recapitulating our cell-based assays, we demonstrated impaired mitochondrial respiration in pancreatic mouse islets isolated from *Atm*^*−/−*^ mice, a function that is essential for pancreatic islet insulin release^59^.

Last, we supplemented our *Atm*^*−/−*^ mice with NAC water. Remarkably, NAC treatment prevented both the glucose response defect and the accumulation of hepatic lipids. Furthermore, NAC treatment rescued the glutamate accumulation in the pancreatic islets, presumably by reducing intracellular glutamate via GSH production. These data strongly support the use of NAC as an effective treatment for A-T patients, where other antioxidants would fail as they would not deplete intracellular glutamate levels. It should be noted that depleted GSH pools in the absence of ATM were reported more than 20 years ago in A-T fibroblasts and red blood cells^60,61^. Similarly, a more recent study, using cerebellar astroglia isolated from ATM mutant mice, noted a GSH-homeostasis defect caused by an impaired ability to import cystine^62^. It was assumed this decrease was due to a downregulation of xCT levels, a component of the x_c_^−^ transporter system. It will be interesting to determine if the mechanism we identified is also the basis of their observed defect in that tissue, as this could relate to the ataxia phenotype of A-T.

CD98HC heterodimerizes with numerous partner proteins to both form antiport channels and facilitate their surface localization^63^. We showed ATM impacts the activity of at least two of these antiport channels through CD98HC phosphorylation and given the known functions of additional such channels, it seems plausible that ATM would similarly impact other antiport channels as a novel explanation for at least some of the chronic progressive phenotypes associated with A-T. For instance, i) lymphocyte proliferation, which depends upon CD98/x_c_- activity (A-T patients are immunodeficient due to a lack of T-cells); ii) ataxia, as mutations in several CD98HC partner proteins result in ataxia (mutation of SLC7A9 or SLC7A10 results in ataxia, while SLC7A8 mutation results in impaired motor performance, and the few SLC7A7-null mice that survive have tremors^64–68^); iii) ASC-1, another antiport channel involving CD98HC, participates in L-serine uptake and is important for Purkinje cell survival and dendrite growth, a well-known consequence of A-T; and iv) growth, as y^+^L transports thyroid hormone through the blood-brain barrier (A-T patients often have slightly reduced stature). Overall, our findings indicate novel directions to explore that may lead to effective interventions to treat this syndrome. Finally, considering that ATM is frequently mutated, deleted, or methylated in various cancers, a defect in amino acid (cysteine/glutamate) metabolism may also offer new therapeutic targets that could be explored in those contexts.

## Methods

### Experimental model and subject details

#### Mice

C57BL/6J *Atm*^*+/−*^ (*Atm*^*tm1Awb*^) mice were described previously^31,70^. These mice were backcrossed onto C57BL/6J p^un/un^ mice for more than fifteen generations^70^. Genotypes for the *Atm* allele were determined by PCR amplification as described in^57^. The mice were housed in a pathogen-free barrier facility as approved by UT Health San Antonio (UTHealth) IACUC policy as outlined in our protocol number 07005x. The facility is operated in compliance with Public Law 89–544 (Animal Welfare Act) and its amendments, Public Health Services Policy on Humane Care and Use of Laboratory Animals (PHS Policy) using the Guide for the Care and Use of Laboratory Animals (Guide) as the basis of operation. The University has been accredited by the Association for Assessment and Accreditation of Laboratory Animal Care, International (AAALAC). *Atm*^*+/−*^ female mice were crossed with *Atm*^*+/−*^ males and were given free access to drinking water with or without 40 mM NAC (pH 7.0–7.4, Sigma) throughout pregnancy, lactation, and thence upon weaning. Both, regular and NAC supplemented water were changed weekly. After weaning, the treatment group received the same 40 mM NAC supplemented drinking water until 6 months of age when tests were carried out. All mice were maintained on a normal diet.

#### Cell culture

Three different isolates of Human Umbilical Vein Endothelial cells (HUVEC) were purchased from GIBCO^™^. Cells between passages 3–7 were used for experiments. Cells were grown in Medium 200 (GIBCO^™^) supplemented with 2% Low Serum Growth Supplement (GIBCO^™^) and 1% PS. Three days before the experiments medium was progressively changed to Medium 199 (Earle’s Salts, GIBCO^™^) supplemented with 1mM sodium pyruvate, 1.82mM glutamine, 10mM HEPES, 2% LSGS, 8% FBS, and 1% PS (to keep physiologically relevant glucose and glutamine levels, 5.5mM, and 2mM respectively). 16–20h before experiments, cells were washed twice with HBSS (Corning) and the medium was changed to low serum (2% LSGS only). HUVECs were kept at 3% oxygen and 37 °C in a humidified atmosphere with 5% CO_2_.

Primary MEFs were obtained by intercrossing *Atm* heterozygous mice to obtain *Atm*^*−/−*^ embryos and littermate controls and isolated as previously described^71^. MEFs were grown in DMEM (10–013-CV, Corning) supplemented with 10% FBS, and 1% PS and kept at 3% oxygen.

Mouse aTC1 Clone 9 pancreatic cells, Mouse b-TC6 pancreatic cells, and HEK-293 cells were obtained from ATCC and grown in DMEM supplemented with 10% FBS, and 1% PS. Alpha pancreatic cells medium was further supplemented with 0.02% BSA, 100μM non-essential amino acids, and 10mM HEPES. Three days before experiments the medium was changed to Basal Medium Eagle (BME, GIBCO^™^) containing 10% FBS, 5.5mM Glucose, 1mM Sodium Pyruvate, and 2mM Glutamine. For alpha cells, 0.02% BSA, 100μM NEAA, and 10mM HEPES were also added. 16–20h before experiments, cells were washed twice with HBSS and media changed to 2% FBS-BME. Alpha, beta and HEK-293 cells were maintained at 37°C in a humidified atmosphere with 5% CO_2_ and tested for mycoplasma contamination.

### Method details

#### Metabolic assays

Mitochondrial respiration and glycolytic function were assayed by measuring the oxygen consumption rate (OCR) and extracellular acidification rate (ECAR) in HUVEC, alpha, and beta pancreatic cells using a Seahorse XFe96 Analyzer (Agilent Technologies). Experiments were run using XF Base Medium Minimal DMEM (Agilent Technologies) supplemented with 1mM Sodium pyruvate, 5.5mM Glucose, and 2mM Glutamine (pH 7.4). Injections were prepared following the manufacturer’s protocol for Mito Stress Kit and Glycolysis Stress kit (Agilent technologies). For the modified Mito Fuel Flex test, the first injection consisted of the drug targeting either glucose, glutamine, or fatty acids oxidation followed by regular injections in the Mito Stress Kit. OCR/ECAR were normalized to confluence using an IncuCyteaZOOM phase-only processing module (essenBioscience). Each condition was tested with at least 8 technical replicates and the overall experiment was repeated at least twice for independent validation.

#### ROS levels analysis

Intracellular ROS levels were assayed by ROS-Glo^™^ H_2_O_2_ Assay (Promega) and CellROX^â^ oxidative stress assay (Life Technologies). For ROS-Glo^™^, HUVECs were treated with ATM inhibitors (10μM KU55933/ 5μM KU60019, Apexbio) for 8 hours and luminescence was measured following the manufacturer’s protocol. For CellROX assay, cells were treated with KU60019 for 8, 24, and 48 hours. Treatment with stabilized H_2_O_2_ (200μM, Sigma) was used as a positive control of ROS generation. One hour before collecting cells, they were stained with 5μM of CellROX^â^ Reagent adding it directly to the media. Cells were then washed with 1X PBS, trypsinized, and fixed with 4% paraformaldehyde (PFA). Cells were analyzed with the cell analyzer BD LSRFortessa^™^ X-20. Each condition was tested with technical triplicates and the overall experiment was repeated at least three times for independent validation.

#### Viability assays

Cells were seeded at 30% confluence in 96- or 384-well plates. The next day, cells were treated with the inhibitors (5μM KU60019, 10μM KU55933, and 5μM Erastin (Sigma)) and cell viability was evaluated after 96 h using CellTiter-Glo. Confluency curves were generated using the IncuCyteaZOOM phase-only processing module (essenBioscience). Each condition was tested with technical quadruplicates and the overall experiment was repeated at least three times for independent validation.

#### Stable Isotope Resolved Metabolomics (SIRM) experiments

HUVECs were seeded in 100 mm plates using supplemented M199 medium (Mybiosource.com). On the day of the experiment, the medium was changed to one containing either 5.5mM [U-^13^C]- glucose and unlabeled Gln or 2mM [U-13C,15N]-glutamine with unlabeled glucose and treated with 10μM KU55933 for 8 h. Isotope-enriched substrates were purchased as dry powders from Cambridge Isotope Laboratories, MA. Polar and non-polar metabolites were extracted from cells and media following metabolic quenching in cold acetonitrile and harvesting for metabolite extraction as described previously^72^. Each condition was tested with technical duplicates and the overall experiment was repeated three times for independent validation.

The polar extracts were reconstituted in nanopure water before analysis on a Dionex ICS-5000+ ion chromatography interfaced to a Thermo Fusion Orbitrap Tribrid mass spectrometer (Thermo Fisher Scientific) as previously described^73^ using a m/z scan range of 80–700. Peak areas were integrated and exported to Excel via the Thermo TraceFinder (version 3.3) software package before natural abundance correction^74^. The isotopologue distributions of metabolites were calculated as the mole fractions as previously described^75^. The number of moles of each metabolite was determined by calibrating the natural abundance-corrected signal against that of authentic external standards. The amount was normalized to the amount of extracted protein and is reported in nmol/mg protein.

Polar extracts reconstituted in D_2_O (> 99.9%, Cambridge Isotope Laboratories, MA) containing 17.5nmol d6-2,2-dimethyl-2-silapentane-5-sulfonate (DSS) as internal standard were analyzed by 1D ^1^H and ^1^H{^13^C}-HSQC NMR on a 14.1 T DD2 NMR spectrometer (Agilent Technologies, CA). 1D 1H spectra were acquired using the standard PRESAT pulse sequence with 512 transients, 16384 data points, 12 ppm spectral width, an acquisition time of 2 s and a 6 s recycle time with weak irradiation on the residual HOD signal during the relaxation delay. The raw fids were zero filled to 131072 points and apodized with 1 Hz exponential line broadening prior to Fourier transformation. 1D HSQC spectra were recorded with an acquisition time of 0.25 s with GARP decoupling and recycle time of 2 s over a spectral width of 12 ppm, with, 1024 transients. The HSQC spectra were then apodized with unshifted Gaussian function and 4 Hz exponential line broadening and zero filled to 16k data points before Fourier transformation. Metabolites were assigned by comparison with in-house^76^ and public NMR databases. Metabolite and their ^13^C isotopomers were quantified using the MesReNova software (Mestrelab, Santiago de Compostela, Spain) by peak deconvolution. The peak intensities of metabolites obtained were converted into nmoles by calibration against the peak intensity of DSS (27.5 nmoles) at 0 ppm for ^1^H spectra and that of phosphocholine at 3.21 ppm (nmoles determined from 1D ^1^H spectra) for HSQC spectra before normalization with mg protein in each sample.

#### Metabolite analysis

Intra- and extra-cellular levels of glutamate were measured with Glutamate assay Kit (Sigma-Aldrich) and Amplex Red Glutamic Acid Assay kit (Molecular probes) respectively. For each experiment, cells were seeded in 6-well plates (HUVECs) and 12 well plates (alpha and beta pancreatic cells) and treated with either 10μM KU55933 or 5μM KU60019 for 8h. Harvesting and measurements were performed following manufacturers’ protocol. Values were normalized to protein concentration. Each condition was tested with technical duplicates and the experiments were repeated three times. Both, GSH-Glo^™^ Glutathione Assay and GSH/GSSG-Glo^™^ Assay (Promega) were used to measure total, reduced, and oxidized GSH after drug treatments. NADP/NADPH-Glo^™^ Assay (Promega) was performed in HUVECs following the manufacturer’s protocol. Each condition was tested with technical duplicates and the experiments were repeated twice for independent validation.

#### Total RNA isolation and sequencing

HUVECs were treated with either 10μM KU55933 or 5μM KU60019 for 8h. RNA was isolated using the RNeasy Mini Kit (Qiagen). Approximately 500ng Total RNA was used for RNA-seq library preparation by following the Illumina TruSeq stranded mRNA sample preparation guide. The first step in the workflow involves purifying the poly-A-containing mRNA molecules using poly-T oligo-attached magnetic beads.

Following purification, the mRNA is fragmented using divalent cations under elevated temperatures. The cleaved RNA fragments are copied into first strand cDNA using reverse transcriptase and random primers. This is followed by second strand cDNA synthesis using DNA Polymerase I and RNase H. Strand specificity is achieved by replacing dTTP with dUTP in the Second Strand Marking Mix (SMM). These cDNA fragments then go through an end repair process, the addition of a single ‘A’ base, and then ligation of the adapters. The products are then purified and enriched with PCR to create the final RNA-seq library. After RNA-seq libraries were subjected to quantification process, pooled for cBot amplification and subsequent 50bp single read sequencing run with Illumina HiSeq 3000 platform. After the sequencing run, demultiplexing with Bcl2fastq2 was employed to generate the fastq file for each sample with about 35–40 Mreads per sample.

#### Lentiviral infection

Lentiviral production was performed following previously published work^77^. Briefly, HEK-293 cells were co-transfected with packaging plasmids pM2.G and psPAX2 (Addgene) and ATM shRNA or Control shRNA (Santacruz) using Lipofectamine 2000 (Life Technologies). After 8 hours, the medium was changed and supplemented with 0.5% bovine serum albumin (Sigma) to improve virus stability. After 60 hours, viral supernatants were recollected, centrifuged at 300g at 4°C for 10 min, and filtered through a 0.45μm low protein binding membrane (Millipore). Single-use aliquots were made and stored at −80°C. HUVEC, alpha, and beta pancreatic cells were transduced with the Lentiviral particles when they reached 70% confluence. 10μg/mL Polybrene was added to improve transduction efficiency and the medium was changed after 8 hours. The next day, the medium was supplemented with 1μg/mL puromycin (GIBCO^™^). Cells were growing with puromycin for at least 5 days before running experiments. Successful transduction was confirmed by the detection of ATM (Sigma) by western blot.

#### Immunofluorescence and proximity ligation assays (PLA)

Cells were plated in coverslips pre-coated with 1% gelatin. HUVECs were treated with 10μM KU55933 for 8 hours and addition of 200μM H_2_O_2_ for the last 2 hours. Cells were fixed with 4% PFA and permeabilized with 0.5% Tween in PBS at room temperature (RT). After incubation in blocking buffer (5% bovine serum albumin in PBS), primary antibody (Rabbit anti-ATMphospho S1981) was added for 4h at RT in a moist chamber. Secondary antibody (Alexa Flour donkey anti-rabbit 488 IgG) and Hoechst 33342, were added for 1 h, and cells were washed and covered with Prolong Gold antifade reagent (Thermo Fisher). For the PLA (Sigma), cells were either treated with 5μM KU60019 (8h) or transduced with shATM lentiviral particles (Santa Cruz Biotechnologies) beforehand. The PLA was performed following the manufacturer’s protocol. Primary antibodies used were goat anti-ATM (Sigma) and rabbit anti-CD98 (Cell signaling). Images were recorded on a Confocal Laser Scanning Microscope (Olympus FV3000). Fluorescent images were acquired in a scan format of 1024 × 1024 pixels in a spatial data set (xyz) and were processed with Image J software. Controls without primary antibodies showed no fluorescence labeling.

#### Immunoblotting and immunoprecipitation

Besides ATM inhibitors, HUVECs were treated with ATR (2μM AZD6738), DNAPKs (1μM AZD7648), AKT (3μM MK2206) inhibitors for 8 hours and 200μM H_2_O_2_ was added for the last 2 hours. Whole-cell lysates were prepared using NaCl Lysis buffer (20mM Tris-HCl (pH 7.5), 150mM NaCl, 1mM Na_2_EDTA, 1mM EGTA, 1% Triton, 2.5mM sodium phosphate, 1mM ß-glycerophosphate) containing a proteases inhibitor cocktail (Roche/Sigma) and phosphatase inhibitor (Thermo Fisher) following standard methods. 40–50μg of total protein was loaded in either precast 3–8% gradient gels (Invitrogen) or laboratory-prepared gels and transferred onto a nitrocellulose membrane. All blots were incubated with primary antibodies overnight and developed using enhanced chemiluminescence (ECL, ThermoFisher). Antibodies used in this study include ATMphosphoS1981 (Abcam), ATM (Sigma and Santa Cruz), CD98phosphoS103, CD98 (Cell signaling), CD98 (H-300 Santa cruz), ß-ACTIN (Abcam), GAPDH, LAMIN-2, MEK1/2, VINCULIN, AKTphosphoS473, AKT, and CHK1phosphoS347 (Cell signaling), DNA PKcs-phosphoS2056 and DNAPKs (Abcam). Co-immunoprecipitation experiments were done with endogenous proteins as described before^78^. In brief, cells from nearly confluent 15-cm plates treated with 200μM H_2_O_2_ for 30min were harvested and lysed in IP buffer (20mM HEPES, 150mM NaCl, 2mM MgCl_2_, 0.5mM CaCl_2_, 1% (v/v) Brij-35 and protease inhibitor cocktail). The lysates were incubated at 4°C with gentle rotation for 1 hour, centrifuged at 12,000g for 10 minutes and the supernatants were used for immunoprecipitation. Lysates were pre-cleared using 20μL of protein A/G dynabeads (Invitrogen) for 30 min at 4°C. 650μg total cell lysates were incubated with 4–8 μg of the appropriate primary antibody overnight at 4°C. The next day, 50μL of protein A/G dynabeads were added to the lysates and incubated for 2 hours at 4°C. Beads were washed three times in IP buffer and bound proteins were eluted by boiling the beads in NuPAGE sample buffer under reducing conditions. Eluted proteins were evaluated by immunoblotting and compared to inputs (10% of the amount used for immunoprecipitation). Co-immunoprecipitation experiments were repeated with biological replicates at least three times in independent sample preparations.

To assay the glycosylation status of CD98HC, HEK293T cells were treated with or without tunicamycin (Sigma-Aldrich, #T7765) in regular maintenance medium for 24 h (0.5 and 1 ug/mL) or 8 h (5 ug/mL). Cells were then harvested and lysed in IP buffer and western blots were performed as previously described.

#### [^14^C]-cystine and -arginine uptake

Amino acid uptake assays were performed as previously described with some minor modifications^79,80^. Briefly, HUVECs were seeded in 12-well plates. When they reached nearly 90% confluence 8h treatments were initiated adding DMSO, 10μM KU55933, 5μM KU60019, or 400μM Sulphasalazine (SAS). For ATM+/+ and ATM−/− derived MEFs, cells were seeded in two 24-well plates and left to grow for about 40h when SAS treatment was started. 4h later, the medium in both plates was removed and HBSS was added during the ionizing radiation treatment (0.5Gy) using the Faxitron X-ray 43855F (Bookholt Associates). Cells were fed with fresh medium and returned to the incubator for 1 hour before harvesting. At the end of the treatments, cells were washed three times with pre-warmed (37°C) transport medium (137mM NaCl, 0.7mM K_2_HPO_4_, 1mM CaCl_2_, 1mM MgCl_2_, 5mM glucose, 10mM HEPES, pH 7.4). Then, the transport medium was replaced with 400 μL transport buffer containing treatments and [^14^C] L-cystine (PerkinElmer) or [^14^C] Arginine (PerkinElmer) at a final concentration of 5μM and 20μM/well respectively. Cells were incubated at 37°C for 10 min. Amino acid transport was stopped by placing the plate on ice. HUVECs were then washed three times with ice-cold transport medium and lysed in 200μL 0.1M NaOH. Radioactivity in lysates was measured by liquid scintillation counting and normalized to the quantity of protein in lysates as determined using the Pierce BCA (bicinchoninic acid) Protein Assay kit (Thermo Fisher). Experiments were performed in technical triplicates and repeated three times for independent validation.

#### CD98 PhosphoS103 monoclonal antibody production and validation

Bioinformatics analysis of CD98HC identified a putative site in its cytoplasmic tail (CGTM**SQ**DTEVDMK) which was previously reported to be phosphorylated in prior phosphoproteome screens for ATM-dependent phosphorylation events^81^. The project was submitted to BIOMATIK to generate the respective phospho-specific antibody. Validation of CD98phosphoS103 antibody was done by showing the specificity of designed primers (dot bot) and by treating lysates with alkaline phosphatase, Calf Intestinal (CIP, New England Biolabs) followed by immunoblotting. For the first approach, designed phospho peptides (Biomatik) were diluted into 5, 10 and 20μg/mL in PBS (pH7.4). 100μL per dilution was loaded onto a pre-wet H+ nylon membrane. The membrane was washed twice with dH2O and then left to air dry at room temperature. The membrane was then blocked with 1X TBST containing 5% non-fat dry milk for 1h and incubated with our customized primary antibody for CD98phosphoS103 (Biomatik) in the blocking buffer for 2.5h. Anti-mouse IgG-HRP was incubated for 45 min and blots were developed using enhanced chemiluminescence (ECL, ThermoFisher) and the Odyssey^®^ FC imaging system (LI-COR Biosciences).

#### Subcellular fractionation

Subcellular fractions were purified using NE-PER Nuclear and Cytoplasmic Extraction Reagents (Thermo Scientific). HUVECs were seeded in 10cm plates and treated with 200μM H_2_O_2_ for 2 hours. Cells were harvested following the manufacturer’s procedure. Enriched fractions were blotted against anti-CD98phosphoS103, CD98 (Cell Signaling), pATM (Abcam), ATM (Sigma-Aldrich), xCT (Abcam), LAT-2 (Abcam). Anti-LaminB1 and MEK1/2 (Cell Signaling) were used as nuclear and cytoplasmic markers respectively. Anti-CD98 (Cell Signaling) was used as a membrane marker. Blots were developed using enhanced chemiluminescence (ECL, ThermoFisher) and the Odyssey^®^ FC imaging system (LI-COR Biosciences). The experiment was performed three times for independent validation.

#### Photoconversion assay for in vivo protein tracking

First, to create the phospho-dead (PD) mutant, the A and G at positions 307 and 308 of SLC3A2 were mutated to G and C respectively which converted the serine to alanine. Mutagenesis was performed in the plasmid CD98 (SLC3A2) (NM_002394) Human Tagged ORF Clone (Origene, RG216640) using the QuickChange II sytem (Qiagen), the primers used were: acctcggtgtcctgggccatggtgcctgtaac and gttacaggcaccatggcccaggacaccgaggt. Positive clones were verified by Sanger sequencing (Genewiz, Azenta Life Sciences).

Next, the photoconvertible protein mEos3.2 (kindly provided by Dr. Lechleiter) was inserted in the N-terminus of both wild-type and phospho-dead SLC3A2 CDS by PCR using the following primers: NM_002394_F: atggagctacagcctcctgaagcctcgatc; PmeI_Stop_NM_002394_R: cgcggccggccgtttaggccgcgtaggggaagcggagcagcagccc. The fragment coding for mEos3.2 (mEos3.2_1xHis_STOP_pET28a,) was extracted using the following primers: BamHI_mEos32_F: ttcgtcgactggatcatgagtgcgattaagccagacatgaagatc; NM_002394_mEos32_R: aggctgtagctccattcgtctggcattgtcaggcaatccagaatg. Next, SLC3A2 and GFP were removed from the Origene plasmid using BamHI-HF (NEB, R3136) and PmeI (NEB, R0560) double enzyme digest, and this backbone was used to insert the two PCR products (SLC3A2 and mEos3.2) using the In-fusion HD Cloning Kit (Takara # 638909).

Positive clones were verified by Sanger sequencing (Genewiz, Azenta Life Sciences).

For the transfection and photoconversion, HEK293T cells were cultured on 35 mm glass bottom plates (MatTek # P35GC1.514C) and transfected with 1 μg of wild-type or phospho-dead plasmid using Lipofectamine 3000 (Invitrogen). 24 hours after transfection, cells were exposed to a 405nm laser for 3 minutes to photoconvert mEos3.2, which normally emits green fluorescence and after exposure will also emit red fluorescence. Cells were treated with CellBritea Steady Membrane Staining (Biotium) and images from live cells were captured 24 hours after the photoconversion on an Olympus FLUOVIEWO FV3000 confocal microscope.

#### Angiogenesis and wound healing assays

24 well plates were coated with 92μL of Geltrex^®^Matrix and incubated at 37°C for 30 min. HUVECs were pre-treated with 5μM KU60019, 2mM NAC, or 5mM L-Arginine ethyl ester (LAEE, Sigma) for 4 hours. The angiogenesis assay was initiated by seeding 75,000 cells/well in the pre-coated plates and left to form endothelial vessel networks over 16 hours maintaining respective drug treatments. Images were taken using the IncuCyteaZOOM phase-only processing module (essenBioscience). Images were processed with the software Image J and analyzed with the angiogenesis tool. The selected parameters were: Number of meshes; Total master segment length, the sum of the length of the detected master segments; Mesh index, the mean distance separating two master junctions in the trees; Number of nodes; and Number of master segments. 20 images were analyzed per condition. Experiments were performed in duplicate and repeated twice for independent validation. For the wound healing assay, cells were seeded the day before and scratched performed using the WoundMaker^™^ instrument (essenBiosience). Cells were washed twice with HBSS and treatments were initiated at this point. Images were taken after 12h using the IncuCyteaZOOM and quantified using Image J. Experiment was done twice in quadruplicates.

#### Insulin and glucagon ELISA

Alpha and beta pancreatic cells were seeded in 96 well plates and treated with either ATM inhibitor. ATM null cells obtained from lentiviral transduction were also used in this experiment. Insulin secretion by beta cells was measured using the Mouse Ultrasensitive Insulin ELISA (ALPCO Diagnostics). Glucagon secretion was measured by using the Quantikine^®^ELISA-Glucagon (R&D Systems) following the manufacturer’s instructions. Briefly, after treatments supernatant was recollected and centrifugated at 300g at 4°C for 10 min to eliminate cell debris. Dilutions of 1:100 and 1:10 were used for insulin and glucagon kits, respectively. The cell lysate was collected in lysis buffer and used to normalize values to protein concentration. Experiments were performed in technical quadruplicates and repeated three times for independent validation.

#### Pancreatic islet isolation and islet bioenergetics

Islet isolation was described previously^82^. Briefly, islets were isolated from 6-month-old C57BL/6J *Atm*^*+/+*^ and *Atm*^*−*/*−*^ mice by collagenase XI (Sigma-Aldrich) perfusion and Histopaque (Sigma-Aldrich) separation from acinar and ductal tissues. Islets were then handpicked and cultured overnight in RPMI 1640 plus 10% FBS at 37°C and 5% CO_2_ before performing assays. Glucose response and bioenergetic studies were performed with pooled islets (50–80 islets/well), using a Seahorse XF24 Analyzer (Agilent Technologies) as previously described^83^. At the end of the experiment, islets were lysed in 30 μL of lysis buffer with a protease inhibitor cocktail (Roche/Sigma), and protein concentration was measured using the BCA protein assay (Thermo Fisher). Protein content was used to normalize seahorse OCR. Each experimental group had 4–5 animals with 2–4 technical replicates.

#### Glucose and insulin tolerance test

Mice were kept on a normal chow diet and underwent glucose and insulin tolerance tests at 6 (males) or 12 months (females) of age. Mice were fasted for 14 hours and injected intraperitoneally with either glucose (2g/kg) or insulin (0.75U/kg). For the oral glucose tolerance test mice were fed 45% glucose solution via oral gavage. Plasma concentrations of insulin were measured using the Mouse Ultrasensitive Insulin ELISA (ALPCO Diagnostics). Tail vein blood glucose was measured using an automated glucometer (Bionime/CVS Glucose Meter) at indicated times. Each experimental group had 5–8 animals.

#### Immunohistochemistry and Oil Red O staining

Mice pancreas were fixed in 10% formalin and embedded in paraffin. 4μm-thick sections were deparaffinized, rehydrated, and treated either with 1mM EDTA pH 8 for 40 min at 95°C followed by a 20-min cool-down step or with citrate pH 6 for 30 min at 98–100°C followed by a 30-min cool-down step. Slides were then rinsed in 1X Tris-buffered saline (TBS) three times. Following endogenous peroxidase blocking, the slides were incubated with primary antibodies (Glutamate (LS Bio), glutamine (Abcam), glucagon, insulin, and CD98 (Cell signaling)) for 2 h at room temperature in a moist humidity chamber. Biotinylated anti-rabbit secondary antibodies (1:200, Vector Laboratories) were incubated for 60 min at room temperature after slides were washed in 1X TBS three times. Slides were incubated in ABC-HRP complex (Vector Laboratories) for 30 min. To confirm antibody specificity, one slide was incubated with secondary antibody only. Slides were then developed with DAB for 5 min, rinsed with TBS, counterstained with hematoxylin, dehydrated, cleared, and mounted with a synthetic mounting medium. Oil Red O staining was performed in frozen livers from 6-month-old C57BL/6J *Atm*^*+/−*^ mice, 4–5 month-old 129Sv/C57B6 *Atm*^*+/−*^ (kind gift of Zha Lab), and Ataxia Telangiectasia donors (University of Maryland Brain & Tissue Bank). Images were taken on a Motic Digital Slide Scanning System at 20X and 40X magnification.

### Quantification and Statistical analysis

#### Photoconversion image analysis and quantification

CD98HC protein localization to the membrane was measured by taking three cross sections per cell using the straight-line feature in ImageJ, spanning the length of the cell. CD98HC fluorescence signal corresponding to the peak membrane fluorescence was extracted from the ImageJ plot profiles of each of the three cross sections. The grey values across the pixel distance of each cross-section were binned into 100 equal parts using RStudio. The mean fluorescence intensity from all three cross sections was averaged for each bin for a total of 100 normalized fluorescence intensity values per cell. The CD98HC membrane signal corresponded to bins 1–5 and 95–100, and the intracellular signal to bins 6–94.

#### Immunohistochemistry quantification

Image J software was used to assess microscopic analysis. First, the whole tissue section was exported, and hematoxylin staining was separated using color deconvolution. Primary antibody intensity was measured in each pancreatic islet (average intensity/islet area) and normalized to the surrounding background. To measure islet size, insulin staining was used to identify the islets. Glucagon staining was quantified in each islet and expressed as a percentage of positive area/pancreatic islet area. Oil Red O (ORO) staining was quantified and expressed as a percentage of the positive area in the entire pancreatic section.

#### Statistics

Data analyses were performed using GraphPad Prism 9.4.1. p-values were provided in the corresponding figure legend and calculated using one- or two-way ANOVA. For non-parametric data, Mann-Whitney or Kruskal-Wallis tests were performed. p<0.05 was considered significant: *p<0.05, **p<0.01, ***p<0.001, ****p<0.0001. When not specified, asterisks show significance vs. control. All *in vitro* experiments were repeated at least three times with 2–4 technical replicates unless specified otherwise in the figure legends. Mice were randomly assigned for the *in vivo* studies; the sample size is provided in the figure legends.

### Bioinformatics analysis

#### Correlation Analysis

Re-processed RNA-Sequencing counts and metadata were generated by the authors of the ARCHS4 repository as described in their recent publication^84^. Counts and metadata were downloaded from the *ARCHS4* repository and pre-processed with custom R scripts. Samples were categorized using a manually curated dictionary of disease-related regex terms, allowing isolation of normal tissue samples. Count data filtering followed a five-step procedure: (1) scRNA-Seq samples were identified using a custom regex dictionary and removed because of the demonstrated unsuitability of single-cell data for co-expression network inference by Pearson correlation^85^. (2) Samples with fewer than 5 million raw read counts were discarded to improve the quality of our gene co-expression calculations by reducing noise from low-quality samples^86^. (3) Genes with zero raw counts in 10% or more of samples were removed to further reduce noise; an approach based on the recommendations of *WGCNA* authors^87^. (4) For each tissue-disease group, studies with only one sample were removed due to the inability to calculate batch effects in these samples^88^. (5) Tissue-disease groups with fewer than 30 distinct samples or with fewer than 4 different studies by GEO series accession (GSE) were removed to limit the effects of bias from individual samples or studies and improve the performance of co-expression calculations^86^. Count data was normalized and batch-corrected following the procedure outlined by the ARCHS4 authors^84^: Raw counts were log2(x+1) transformed and subsequently quantile normalized using the *normalize.quantiles* function from the *preprocessCore* R package^89^. Then, batch effects were removed based on a study with the *ComBat* function of the *sva* R package^88^. Then, gene-gene Pearson correlations were calculated using the *cor* function of the *WGCNA* package^87^. Correlations for *ATM* were used as the ranking criteria for Gene Set Enrichment Analysis (GSEA) implemented via the *Cluster Profiler R* package’s *GSEA* function and visualized with their *gseaplot* function using annotations gathered via the *msigdbr* package’s *msigdbr* function^90,91^.

#### Single-cell RNA-Seq of Pancreas Tissue

A comprehensive single-cell RNA-Seq dataset of human pancreas tissue was generated via the analysis methods described by the authors of *Seurat* in their data integration guide using the data objects conveniently provided for download on the Seurat web page^92,93^. The data provided for download was derived from three single-cell studies of pancreatic tissue: GSE81076/GSE85241^94^, GSE86 469^95^, and E-MTAB-5061^96^. The expression of *SLC3A2* and *ATM* was plotted across cell types (as was determined by the Seurat authors) in the form of ridge plots using the *RidgePlot* function of Seurat. The expression of *ATM* and *SLC3A2* were quantile normalized using the *normalizee.quantiles* function of the *preprocessCore* package^89^. Then normalized *ATM* and *SLC3A2* expressions were compared using the *stat_compare_means* function of the *ggpubr* package across cell types and plotted using the *ggboxplot* function^97^.

#### Bulk RNA-Seq

Raw data were processed into fastq files using the *bcl2fastq* software program (Illumina). Fastq files were trimmed for adapter sequences using the *fastp* software program^98^. Trimmed fastq files were aligned to the GRCh38 transcriptome (Gencode v30) using the *salmon* aligner software^99^. Read counts were summarized to the gene level using the *tximport* R package^100^. Differential gene expression was calculated using the *DESeq2* R package, and log-transformed p-adjusted values (FDR) were used as the ranking criteria for Gene Set Enrichment Analysis implemented via the *Cluster Profiler* package’s GSEA function and visualized with their *gseaplot* function using annotations gathered via the *msigdbr* package’s *msigdbr* function^90,91^.

## Data Availability

The fastq files and read counts for all bulk RNA-Sequencing runs have been deposited in NCBI GEO under accession GSE140416.
